# Microbiological Characteristics and Predictive Factors for Mortality in Pleural Infection: A Single-Center Cohort Study in Korea

**DOI:** 10.1371/journal.pone.0161280

**Published:** 2016-08-16

**Authors:** Cheol-Kyu Park, Hyoung-Joo Oh, Ha-Young Choi, Hong-Joon Shin, Jung Hwan Lim, In-Jae Oh, Yu-Il Kim, Sung-Chul Lim, Young-Chul Kim, Yong-Soo Kwon

**Affiliations:** Department of Internal Medicine, Chonnam National University Hospital, Gwangju, South Korea; Institut Pasteur, FRANCE

## Abstract

**Background:**

Identification and understanding of the pathogens responsible for pleural infection is critical for appropriate antibiotic treatment. This study sought to determine the microbiological characteristics of pleural infection and to identify potential predictive factors associated with mortality.

**Methods:**

In this retrospective study, we analyzed patient data from 421 cases of parapneumonic effusion. A total of 184 microorganisms were isolated from 164 patients, using two culture systems: a standard method and a method using pairs of aerobic and anaerobic blood culture bottles.

**Results:**

The most frequently isolated microorganisms were streptococci (31.5%), followed by staphylococci (23.4%), gram-negative bacteria (18.5%) and anaerobes (10.3%). Streptococci were the main microorganisms found in standard culture (41.9%) and community-acquired infections (52.2%), and were susceptible to all antimicrobial agents in drug sensitivity testing. Staphylococci were the most frequently isolated pathogens in blood cultures (30.8%) and hospital-acquired infections (38.3%), and were primarily multidrug-resistant (61.8%). In multivariate analysis, the following were significant predictive factors for 30-day mortality among the total population: CURB-65 ≥ 2 (aOR 5.549, 95% CI 2.296–13.407, *p*<0.001), structural lung disease (aOR 2.708, 95% CI 1.346–5.379, *p* = 0.004), PSI risk class IV-V (aOR 4.714, 95% CI 1.530–14.524, *p* = 0.007), no use of intrapleural fibrinolytics (aOR 3.062, 95% CI 1.102–8.511, *p* = 0.014), hospital-acquired infection (aOR 2.205, 95% CI 1.165–4.172, *p* = 0.015), age (aOR 0.964, 95% CI 0.935–0.994, *p* = 0.018), and SOFA score ≥2 (aOR 2.361, 95% CI 1.134–4.916, *p* = 0.022).

**Conclusion:**

In this study, common pathogens causing pleural infection were comparable to previous studies, and consisted of streptococci, staphylococci, and anaerobes. CURB-65 ≥2, structural lung disease, PSI risk class IV-V, no use of intrapleural fibrinolytics, hospital-acquired infection, older age, and SOFA score ≥ 2 are potential predictors of mortality in pleural infection.

## Introduction

Pleural infection is an ancient disease; however, it remains an important clinical problem, with a rising incidence in recent decades [1]. The cause of this increase is unclear. Possible explanations include the following: the emergence of serotypes not covered by pneumococcal vaccination in young adults; the increase incidence of infection in elderly individuals, who have a higher risk of comorbid conditions than young people and could also be susceptible to gram-negative bacteria and *Staphylococcus aureus*; and the increase in new diagnostic techniques for this disease [1].

The appropriate use of antibiotics is an important factor in decreasing the risk of mortality in pleural infection. Therefore, an understanding of bacteriology is critical for the treatment of this disease. However, the pathogens causing pleural infection are different from those causing community-acquired pneumonia; moreover, they may be affected by underlying diseases, such as end-stage renal disease, and may have changed markedly in recent years [2–8]. Moreover, identification of the causative pathogen may be challenging, due to the low isolation rates in standard culture, seen in up to 60% of cases [4, 5, 9]. A recent study showed that performing BACTEC blood culture of infected pleural fluid, in addition to standard culture, increased the isolation rates of bacteria from 38% in standard culture to 59% [5]. However, the isolation rate of pleural pathogens is not sufficient to guide the selection of antibiotics to treat the disease. Therefore, in many cases of pleural infection, proper selection of antibiotics depends on the knowledge of the typical pathogens found in the community.

Studies on pathogens and prognostic factors of pleural infection in adult patients in Asian countries have been reported [3, 7, 10–16], however, there are scarce studies in South Korea and these involve small numbers of subjects[14, 17]. This study aimed to determine the microbiologic characteristics of pleural infection and to identify predictive factors associated with mortality.

## Methods

### Study subjects and data collection

This study retrospectively reviewed the data of 502 patients, aged 18 years and above, who were diagnosed and treated for pleural infection, after thoracentesis or tube thoracostomy, at Chonnam National University Hospital, between January 2008 and June 2014. The following information was searched for and identified from patient electronic medical records: baseline clinical data, comorbidities, severity score of infection (CURB-65, Sequential Organ Failure Assessment [SOFA] score, Pneumonia Severity Index [PSI] score), origin of infection (community- or hospital-acquired), microbiological characteristics (microbiology, type of culture systems, multiplicity of isolates, drug resistance), pleural fluid analysis, medical and surgical treatment, and outcome (30-day mortality).

Inclusion criteria were the presence of complicated parapneumonic effusion with positive cultures or clinical evidence of infection associated with at least one of the following: pleural fluid pH < 7.2, lactate dehydrogenase (LDH) > 1000 IU/L, or glucose < 60 mg/dL [18]. Pleural infections caused by *Mycobacterium tuberculosis* or non-tuberculous mycobacteria and parasites, or those associated with malignant effusion, were excluded. Polymicrobial infections identified in one specimen or in repeated tests were included if pathogens were identified.

A hospital-acquired infection was determined if the onset of pleural infection had occurred over 48 hours after hospitalization, if the patient had been hospitalized within the preceding 4 weeks, or if infection resulted from a complication of invasive thoracic procedures [9].

Multidrug resistance (MDR) was defined as non-susceptibility to at least one agent in three or more antimicrobial categories in drug sensitivity testing [19], with the exception of methicillin-resistant *Staphylococcus aureus* (MRSA), owing to its resistance to all categories of beta-lactam antimicrobials when it shows resistance to oxacillin or cefoxitin [19].

Among therapeutic interventions, drainage included procedures using a pigtail, which is a small-bore chest tube (10–14 French), and tube thoracostomy, which is a large-bore chest tube (more than 24 French). Urokinase was administered as an intrapleural fibrinolytic agent.

All patients commenced antibiotics according to the 2003 and 2010 British thoracic society (BTS) guidelines for the management of pleural infection [6, 20]. In cases of suspected resistant organism, such as hospital-acquired empyema, extended spectrum antibiotics, combined with glycopeptides, were added for coverage of MRSA [6]. Initial empirical antibiotic regimes were changed according to isolated organisms in culture positive cases. The prescribing physician complied with standard antibiotic dosage regulations and practice was monitored by the infection control unit in this medical center. Antibiotic treatment was regarded as concordant if the organisms appeared sensitive, according to susceptibility testing in culture-positive specimens [11]. Antibiotic therapy was considered discordant if the organisms were resistant to treatment [11]. The choice of interventions was followed by treatment guidelines for pleural infection [6, 21]. Intrapleural fibrinolytics were instilled if there was no evidence of radiological and/or clinical response to antibiotics. If there was no clinical and/or radiological response to intrapleural fibrinolytics, then surgery was performed.

### Pleural fluid collection and culture systems

When pleural fluid was detected on imaging, a 20 ml sample was obtained by thoracentesis under aseptic conditions. After the evaluation of the general appearance of fluid, the sample was processed by standard body fluid culture, a blood culture system, or both methods. In our institute, we used both culture methods after January 2011. Where blood cultures were used, the sample was injected into pairs of aerobic and anaerobic blood culture bottles (BACTEC PLUS, Becton Dickinson, Sparks, Maryland, USA). In addition, biochemical pleural fluid analysis was performed to identify characteristics, such as pH, leukocyte, glucose, protein, and LDH.

### Statistical analysis

All data are expressed as medians with an interquartile range (IQR) or a percentage. Between-group comparisons were performed using the Mann-Whitney U test for continuous variables, and Pearson’s χ^2^ test or Fisher`s exact test for categorical variables. When calculating the proportion of each microorganism in whole samples or comparing the categorical variables according to individual isolates, the total number of microorganisms was used for statistics, rather than the number of patients, due to the multiplicity of isolates in polymicrobial cases.

Multivariate analysis was performed to determine predictive factors associated with mortality in patients with pleural infection. Possible predictive factors, found to be significant in univariate analysis, were analyzed by binary logistic regression. Statistical analysis was performed with IBM^®^ SPSS^®^ statistics version 19 (SPSS, Inc., an IBM Company), and p < 0.05 indicated statistical significance.

### Ethics statement

Permission was obtained from the Institutional Review Board of Chonnam National University Hospital to review and publish patient records retrospectively (IRB No. CNUH-2014-243). Informed consent was waived due to the retrospective nature of the study, and patient information was anonymized and de-identified prior to analysis.

## Results

### Baseline clinical and pleural fluid characteristics of study subjects

**Fig 1:**
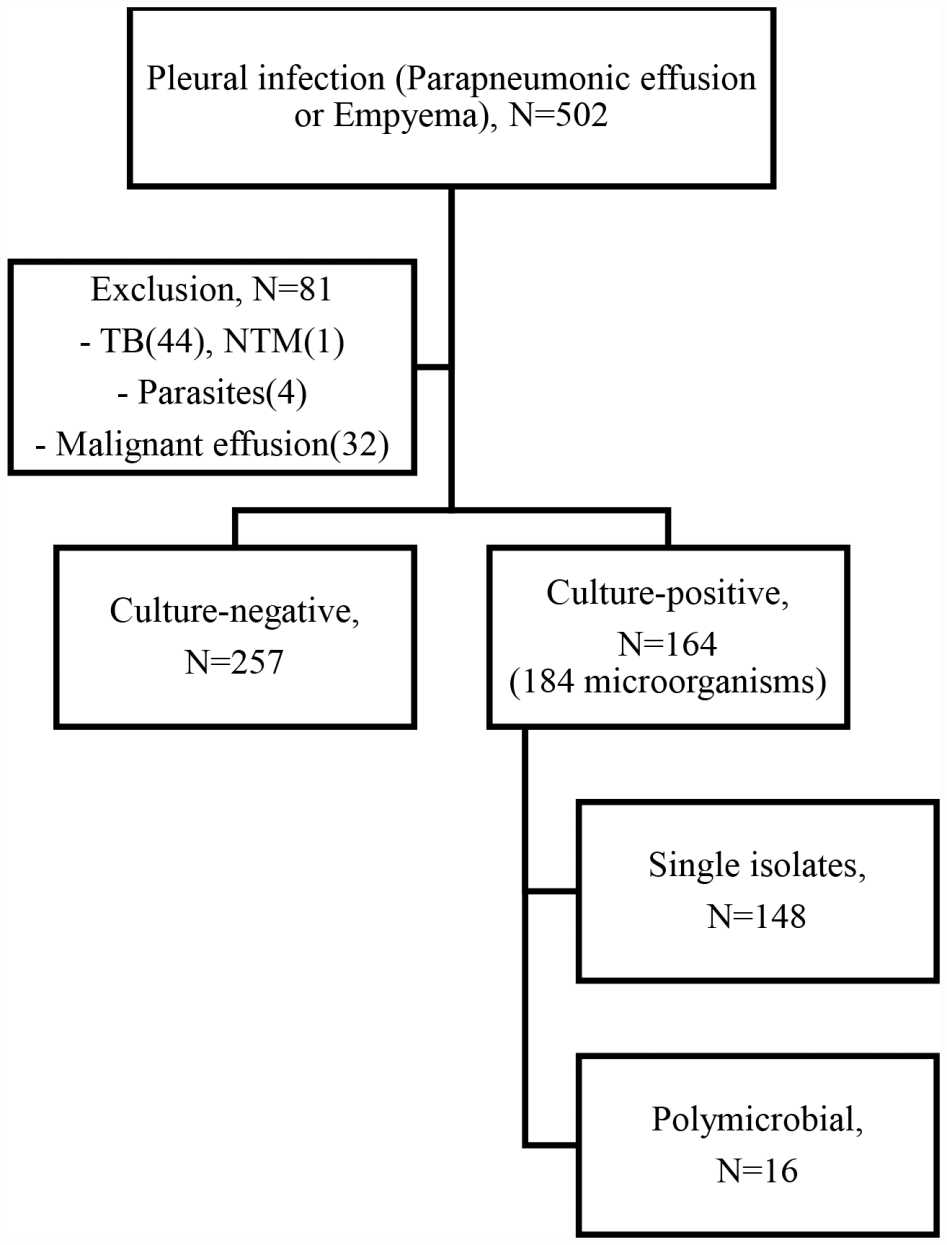
A flowchart of patient enrollment. A total of 502 patients were initially recruited. Following the exclusion of 81 patients, 421 patients were finally included in this study. These were classified into two groups, according to whether cultures were positive or negative. The 164 culture-positive patients were further divided into two groups (single isolates or polymicrobial infection). TB = Tuberculosis, NTM = Nontuberculous mycobacterium.

The algorithm for patient enrollment is shown in Fig 1. A total of 502 patients were initially recruited. Of these, 81 patients were excluded due to infection with *Mycobacterium tuberculosis*, non-tuberculous mycobacteria, or parasites or because of malignant effusion. Therefore, 421 patients were finally included in this study. A total of 184 microorganisms were identified in 164 culture-positive patients.

The median age for study participants was 66 years and the proportion of male participants was 77.9%. Most patients had comorbidities, the majority of which were cardiac or vascular diseases, followed by diabetes and structural lung diseases, including chronic obstructive pulmonary disease, bronchiectasis, interstitial lung disease, and parenchymal destruction associated with old inflammation, such as tuberculosis. Baseline clinical and pleural fluid characteristics of subjects are shown in Table 1.

**Table 1:** Clinical and pleural fluid characteristics in patients with pleural infection.

Variables	Total, n = 421
Age, years	66 (53–74)
Male	328 (77.9)
Comorbid conditions	328 (77.9)
Cardio-cerebrovascular disease	201 (47.7)
^a^Structural lung disease	85 (20.2)
Diabetes	116 (27.6)
Malignancy	39 (9.3)
Chronic kidney disease	47 (10.2)
Chronic liver disease	43 (10.2)
^b^Others	60 (14.2)
Ever smoked, n (%)	234 (55.5)
Pleural fluid characteristics	
Visibly purulent	221 (52.5)
pH	7.02 (6.72–7.16)
Leukocyte, /mm^3^	4295 (1080–24075)
Glucose, mg/dL	36.0 (9.0–91.0)
Protein, g/dL	4.1 (3.0–4.9)
Lactate dehydrogenase, IU/L	2337 (1307–5150)

Data are presented as a percentage (%) or median (interquartile range).

^a^Chronic obstructive pulmonary disease, bronchiectasis, interstitial lung disease, and parenchymal destruction associated with old inflammation, such as tuberculosis.

^b^Anorexia nervosa, connective tissue disease, Crohn’s disease, Cushing syndrome, epilepsy, gout, Graves’ disease, hemolytic uremic syndrome, hypothyroidism, osteoarthritis, pancreatitis, Parkinson’s disease, schizophrenia, and spinal stenosis.

### Culture characteristics and microbiology

A total of 184 culture positive pleural fluid specimens were isolated from 164 patients. In one specimen, there no polymicrobial infection was identified. All specimens were proven by repeated tests. In total, 86 specimens were cultured by standard method only, 65 by the BACTEC culture system only, and 33 samples by both culture systems. Among the 114 cultured specimens using both systems, the BACTEC blood culture system showed a higher culture-positive rate than the standard culture system (97/114 [85.1%] vs. 57/114 [50.0%], *p*<0.001, data not shown here).

Overall microbiology is presented in Table 2. Multiple isolates (≥2) were cultured in 16 patients (9.8%). Of all isolates, the most frequently identified pathogens were streptococci (31.5%), followed by staphylococci (23.4%), gram-negative bacteria (18.5%), and anaerobes (10.3%). The most commonly isolated microorganisms, according to subtype were: *Streptococcus milleri* group, consisting of *Streptococcus intermedius*, *Streptococcus anginosus* and *Streptococcus constellatus;* and *Staphylococcus aureus*. Streptococci were the main microorganisms found in standard culture (41.9%, data not shown in table) and community-acquired infections (52.2%). These organisms were susceptible to all antimicrobial agents in drug sensitivity testing. Staphylococci were the major isolates in BACTEC blood culture (30.8%, data not shown in table), and hospital-acquired infections (38.3%), and were more frequently reported as MDR in drug sensitivity testing (61.8%, data not shown in table). Anaerobes were more frequently identified in BACTEC blood culture (14 vs. 2, *p*<0.001, data not shown in table). Enterococci were generally more drug-resistant (8 vs. 2, p = 0.014, data not shown in table). MDR pathogens were more frequently identified in hospital-acquired infection.

**Table 2:** Comparison of microbiological characteristics between community- and hospital-acquired pleural infections.

Microbiological characteristics	Total (n = 164)	Community-acquired infection (n = 83)	Hospital-acquired infection (n = 81)	p value
Multiplicity of microorganisms				
Single	148 (90.2)	78 (94.0)	70 (86.4)	0.103
Polymicrobial	16 (9.8)	5 (6.0)	11 (13.6)	
Total number of isolated microorganisms	184	90	94	
Aerobes				
*Streptococcus*	58 (31.5)	47 (52.2)	11 (11.7)	<0.001
*S*. *milleri* group (*intermedius/anginosus/constellatus*)	32	26	6	
Viridans streptococci (other than *S*. *milleri*)	13	10	3	
*S*. *pneumoniae*	5	5	0	
*S*. *pyogenes*	2	2	0	
Other *Streptococcus* species	6	4	2	
*Staphylococcus*	43 (23.4)	7 (7.8)	36 (38.3)	<0.001
MRSA	27	1	26	
MSSA	9	4	5	
*S*. *cohnii*	1	1	0	
*S*. *epidermidis*	4	1	3	
*S*. *haemolyticus*	2	0	2	
*Enterococcus* species	10 (5.4)	1 (1.1)	9 (9.6)	0.019
^a^Other Gram positives	10 (5.4)	6 (6.7)	4 (4.3)	0.530
Gram negatives	34 (18.5)	12 (13.3)	22 (23.4)	0.079
*Klebsiella pneumoniae*	17	7	10	
*Acinetobacter baumannii*	5	0	5	
*Enterobacter cloacae*	3	1	2	
*Escherichia coli*	5	3	2	
*Pseudomonas aeruginosa*	4	1	3	
Anaerobes	19 (10.3)	14 (15.6)	5 (5.3)	0.023
*Bacteroides* species	2	1	1	
*Clostridium* species	1	1	0	
*Eggerthella* species	2	0	2	
*Fusobacterium nucleatum*	1	1	0	
*Peptoniphilus asaccharolyticus*	1	1	0	
*Peptostreptococcus* species	9	8	1	
*Prevotella* species	2	2	0	
*Veillonella* species	1	0	1	
Fungus	10 (5.4)	3 (3.3)	7 (7.4)	0.331
*Candida albicans*	4	2	2	
*Candida*, non-*albicans*	4	1	3	
Other *Candida* species	2	0	2	
Isolation of MDR pathogen	55/140 (39.3)	4/63 (6.3)	51/77 (66.2)	<0.001

Data are presented as percentages (%) or median (interquartile range).

^a^*Bacillus*, *Cellulomonas/Microbacterium*, *Corynebacterium*, and *Micrococcus* species.

MRSA = methicillin-resistant *Staphylococcus aureus*; MSSA = methicillin-susceptible *Staphylococcus aureus*.

MDR = Multidrug resistant

Differences between community- and hospital-acquired infection and culture-positive and –negative infection are shown in Table 3. Patients with hospital-acquired pleural infection were more likely to be admitted to ICU, undergo intubation and mechanical ventilation, and have high severity scores (CURB-65≥2, SOFA score ≥2, and PSI risk score IV-V.Culture-positive patients were more likely to have more comorbidities, particularly structural lung disease or chronic liver disease. They were strongly associated with hospital-acquired and high severity scores (CURB-65≥2, SOFA score ≥2, and PSI risk score IV-V). Pleural fluid with culture positive patients showed more leukocytosis.

**Table 3:** Comparisons of baseline and clinical characteristics according to origin of infection and culture status.

Factors	Origin of infection		*p*	Culture status		*p*
	Community, n = 294	Hospital, n = 127		Negative, n = 257	Positive, n = 164	
Age, years	65 (52–75)	66 (57–76)	0.398	65 (52–75)	67 (55–74)	0.615
Male	237 (80.6)	91 (71.7)	0.042	198 (77.0)	130 (79.3)	0.591
Co-morbid conditions	218 (74.1)	110 (86.6)	0.005	192 (74.7)	136 (82.9)	0.047
Cardio-cerebrovascular disease	137 (46.6)	64 (50.4)	0.474	121 (47.1)	80 (48.8)	0.734
^a^Structural lung disease	51 (17.3)	34 (26.8)	0.027	35 (13.6)	50 (30.5)	<0.001
Diabetes	75 (25.5)	41 (32.3)	0.153	65 (25.3)	51 (31.1)	0.194
Malignancy	27 (9.2)	12 (9.4)	0.931	19 (7.4)	20 (12.2)	0.097
Chronic kidney disease	27 (9.2)	20 (15.7)	0.050	25 (9.7)	22 (13.4)	0.241
Chronic liver disease	27 (9.2)	16 (12.6)	0.288	19 (7.4)	24 (14.6)	0.017
Ever smoked	172 (58.5)	62 (48.8)	0.066	142 (55.3)	92 (56.1)	0.865
Hospital-acquired infection	NA	NA	NA	46 (17.9)	81 (49.4)	<0.001
Treatment						
Drainage only	223 (75.9)	84 (66.1)	0.040	190 (73.9)	117 (71.3)	0.560
Intrapleural fibrinolytics	100 (34.0)	25 (19.7)	0.003	79 (30.7)	46 (28.0)	0.556
Surgery	33 (11.2)	16 (12.6)	0.687	26 (10.1)	23 (14.0)	0.223
Antibiotics only	34 (11.6)	25 (19.7)	0.028	38 (14.8)	21 (12.8)	0.568
Positive Culture	83 (28.2)	81 (63.8)	<0.001	NA	NA	NA
Pleural fluid characteristics						
Visibly purulent	176 (59.9)	45 (35.4)	<0.001	144 (56.0)	77 (47.0)	0.069
pH	7.01 (6.70–7.14)	7.12 (6.86–7.28)	<0.001	7.04 (6.82–7.15)	6.95 (6.67–7.24)	0.133
Leukocyte, /mm^3^	5162 (1095–24585)	3440 (893–17165)	0.279	3024 (886–12370)	11900 (2490–66520)	<0.001
Glucose, mg/dL	38.5 (8.5–79.5)	40.0 (10.0–113.0)	0.029	46.0 (10.0–81.0)	29.0 (7.0–97.0)	0.119
Protein, g/dL	4.5 (3.4–5.1)	3.8 (2.9–4.5)	0.023	4.2 (3.1–4.9)	4.0 (2.8–5.0)	0.499
Lactate dehydrogenase, unit/L	2291 (1460–4400)	1962 (1172–5419)	0.056	2223 (1430–4432)	2931 (1181–7776)	0.280
Admission						
Ward	256 (87.1)	92 (72.4)	<0.001	219 (85.2)	129 (78.7)	0.083
ICU	38 (12.9)	35 (27.6)		38 (14.8)	35 (21.3)	
Supplemental Oxygen therapy with nasal prong or mask	184 (62.6)	69 (54.3)	0.019	160 (62.3)	93 (56.7)	0.416
Supplemental Oxygen therapy, L/min, mean ± SD	3.32 ± 0.18	3.23 ± 0.28	0.806	3.39 ± 0.18	3.14 ± 0.25	0.241
Intubation and MV	32 (10.9)	27 (21.3)	0.008	32 (12.5)	27 (16.5)	0.253
MV with high FiO2 (>0.5)	13 (40.6)	9 (34.6)	0.639	8 (25.0)	14 (53.8)	0.024
^b^CURB-65 ≥ 2	121 (41.2)	67 (52.8)	0.028	103 (40.1)	85 (51.8)	0.018
SOFA score ≥ 2	119 (40.5)	64 (50.4)	0.060	122 (47.5)	61 (37.2)	0.038
^c^PSI risk class IV-V	150 (51.0)	91 (71.7)	<0.001	137 (53.3)	104 (63.4)	0.041

Data are presented as percentages (%), median (interquartile range) or mean ± standard deviation (SD).

^a^e.g, chronic obstructive pulmonary disease, bronchiectasis, interstitial lung disease, parenchymal destruction associated with old inflammation, such as tuberculosis.

^b^Scoring system assigns 1 point for each of the following five risk factors: 1) new onset confusion, 2) urea >7 mmol/l (19mg/dL), 3) respiratory rate ≥30 breaths/min, 4) systolic blood pressure <90 mm Hg and/or diastolic blood pressure ≤60 mm Hg and 5) age ≥65 years.

^c^A total point score for a given patient is obtained by adding the patient`s age in years (age minus 10 for women) and the points obtained for each applicable characteristic, such as nursing home resident, coexisting illness, physical examination findings, or laboratory and radiographic findings [22].

NA = Not applicable. ICU = Intensive care unit. MV = Mechanical ventilation. SOFA = Sequential Organ Failure Assessment. PSI = Pneumonia Severity Index.

Initially, empirical antibiotics were administered to all patients. These were subsequently changed according to the organisms isolated in the culture systems. All types of antibiotics used during treatment are shown in Table 4. Third generation cephalosporins or β-lactam/β-lactamase inhibitors combined with clindamycin were the most frequently used antibiotics. Glycopeptides and colistin were used more frequently in hospital-acquired infection. Broad spectrum antibiotics were used more frequently in culture-positive patients.

**Table 4:** Comparisons of antibiotics treatment according to origin of infection and culture status.

Antibiotics, n (%)	Total, n = 421	Origin of infection		*p*	Culture status		*p*
		Community, n = 294	Hospital, n = 127		Negative, n = 257	Positive, N = 164	
^a^Penicillins	4 (1.0)	1 (0.3)	3 (2.4)	0.084	0 (0.0)	4 (2.4)	0.023
^b^Cephalosporins	242 (57.5)	193 (65.6)	49 (38.6)	<0.001	165 (64.2)	77 (47.0)	<0.001
1^st^ generation	24 (5.7)	17 (5.8)	7 (5.5)	0.913	18 (7.0)	6 (3.7)	0.149
2^nd^ generation	1 (0.2)	1 (0.3)	0 (0.0)	1.000	1 (0.4)	0 (0.0)	1.000
3^rd^ generation	214 (50.8)	168 (57.1)	46 (36.2)	<0.001	138 (53.7)	76 (46.3)	0.141
4^th^ generation	17 (4.0)	10 (3.4)	7 (5.5)	0.313	8 (3.1)	9 (5.5)	0.227
^c^β-lactam/β-lactamase inhibitor	112 (26.6)	75 (25.5)	37 (29.1)	0.440	51 (19.8)	61 (37.2)	<0.001
^d^Carbapenems	31 (7.4)	18 (6.1)	13 (10.2)	0.138	12 (4.7)	19 (11.6)	0.008
^e^Aminoglycosides	9 (2.1)	6 (2.0)	3 (2.4)	1.000	8 (3.1)	1 (0.6)	0.163
^f^Fluoroquinolones	30 (7.1)	21 (7.1)	9 (7.1)	0.984	9 (3.5)	21 (12.8)	<0.001
Glycopeptides	36 (8.6)	8 (2.7)	28 (22.0)	<0.001	7 (2.7)	29 (17.7)	<0.001
Vancomycin	22 (5.2)	2 (0.7)	20 (15.7)	<0.001	2 (0.8)	20 (12.2)	<0.001
Teicoplanin	14 (3.3)	6 (2.0)	8 (6.3)	0.036	5 (1.9)	9 (5.5)	0.048
^g^Macrolides	31 (7.4)	30 (10.2)	1 (0.8)	0.001	19 (7.4)	12 (7.3)	0.977
Clindamycin	91 (21.6)	70 (23.8)	21 (16.5)	0.096	57 (22.2)	34 (20.7)	0.725
Metronidazole	52 (12.4)	34 (11.6)	18 (14.2)	0.455	31 (12.1)	21 (12.8)	0.821
Colistin	5 (1.2)	0 (0.0)	5 (3.9)	0.002	1 (0.4)	4 (2.4)	0.078
^h^Others	3 (0.7)	0 (0.0)	0.3 (2.4)	0.027	2 (0.8)	1 (0.6)	1.000

^a^Narrow spectrum; nafcillin

^b^1^st^ generation cephalosporin; ceftezol, cefazedone, cefazolin, 2^nd^ generation cephalosporin; cefbuperazone, 3^rd^ generation cephalosporin; cefditoren, ceftizoxime, cefodizime, cefoperazone, cefotaxime, cefpiramide, ceftazidime, ceftriaxone, flomoxef, 4^th^ generation cephalosporin; cefepime, cefpirome

^c^amoxicillin/clavulanate, ampicillin/sulbactam, tazobactam/piperacillin, ticarcillin/clavulanate

^d^ertapenem, imipenem, meropenem, panipenem

^e^netilmicin, astromicin, amikacin, netromycin, gentamycin, isepamycin

^f^levofloxacin, gemifloxacin, ciprofloxacin, moxifloxacin, ofloxacin

^g^clarithromycin, zithromycin

^h^Trimethoprim/sulfamethoxazole, Anti-tuberculosis medication; isoniazid, rifampin, ethambutol, pyrazinamide, streptomycin

Of 164 culture-positive patients, assessment of the adequacy of antibiotic treatment was feasible in 151 patients (data not shown in the table). Delay in time from admission to initial antibiotic treatment and from admission to change for adequate antibiotics was 8.9 and 24.9 hours in average, respectively. Treatment with initial and appropriately changed antibiotics showed concordance in 118 (78.1%) and 137 (90.7%) patients. Discordant antibiotics treatment (85.5% vs. 95.1%, *p* = 0.042), delay in the use of adequate antibiotics (54.8 vs. 7.8 hours on average, *p*<0.001) and drainage procedure (40.4 vs. 5.5 hours on average, *p* = 0.002) were salient features in patients with hospital-acquired infection. However, these factors were not significantly associated with mortality except a concordance of initial antibiotics (82.5% in survival group vs. 61.3% in death group, p = 0.011) which did not show a significance in multivariate analysis.

#### Predictive factors associated with 30-day mortality

The overall 30-day mortality rate of all patients was 14.3% (60/421). Univariate analysis revealed that the following factors were significant risk factors for 30-day mortality: older age, structural lung disease, hospital-acquired infection, no use of intrapleural fibrinolytics, antibiotic use without intervention, positive cultures, CURB-65 ≥ 2, SOFA score ≥ 2 and PSI risk class IV-V. Isolation of staphylococcus and MDR pathogens, which were more frequent in patients with hospital-acquired infection, were significantly associated with mortality (Table 5). Conversely, no deaths were found in patients with anaerobe infection, more frequently associated with community-acquired infection.

**Table 5:** Univariate analysis for risk factors of 30-day mortality in patients with pleural infection.

Factors	30-day mortality		*p*
	Survival, n = 361	Death, n = 60	
Age, years	65 (53–74)	70 (57–78)	0.032
Male	280 (77.6)	48 (80.8)	0.673
Co-morbid conditions	277 (76.7)	51 (85.0)	0.153
Cardio-cerebrovascular disease	172 (47.6)	29 (48.3)	0.921
^a^Structural lung disease	63 (17.5)	22 (36.7)	0.001
Diabetes	96 (26.6)	20 (33.3)	0.279
Malignancy	31 (8.6)	8 (13.3)	0.240
Chronic kidney disease	38 (10.5)	9 (15.0)	0.308
Chronic liver disease	35 (9.7)	8 (13.3)	0.389
Ever smoked	198 (54.8)	36 (60.6)	0.457
Hospital-acquired infection	96 (26.6)	31 (51.7)	<0.001
Treatment			
Drainage only	259 (71.7)	48 (80.0)	0.183
Intrapleural fibrinolytics	120 (33.2)	5 (8.3)	<0.001
Surgery	46 (12.7)	3 (5.0)	0.083
Antibiotics only	42 (11.6)	17 (28.3)	0.001
Culture positivity	129 (35.7)	35 (58.3)	0.001
Pleural fluid characteristics			
Visibly purulent	194 (53.7)	27 (45.0)	0.209
pH	7.02 (6.76–7.16)	7.09 (6.85–7.19)	0.106
Leukocyte, /mm^3^	4398 (1080–22572)	4680 (792–16040)	0.505
Glucose, mg/dL	36.5 (9.0–81.0)	56.0 (8.5–129.0)	0.128
Protein, g/dL	4.2 (2.9–5.0)	3.8 (3.1–4.8)	0.650
Lactate dehydrogenase, unit/L	2337 (1432–5499)	1772 (570–4787)	0.172
^b^CURB-65 ≥ 2	139 (38.5)	49 (81.7)	<0.001
SOFA score ≥ 2	140 (38.8)	43 (71.7)	<0.001
^c^PSI score risk class IV-V	186 (51.5)	55 (91.7)	<0.001
Microbiology	n = 145	n = 39	
*Streptococcus*	49 (33.8)	9 (23.1)	0.201
*Staphylococcus*	27 (18.6)	16 (41.0)	0.003
*Enterococcus*	9 (6.2)	1 (2.6)	0.691
Other Gram positive organisms	8 (5.5)	2 (5.1)	1.000
Gram negative organisms	25 (17.2)	9 (23.1)	0.405
Anaerobes	19 (13.1)	0 (0.0)	0.015
Fungus	8 (5.5)	2 (5.1)	1.000
Isolation of MDR pathogen	36/107 (33.6)	19/33 (57.6)	0.014

Data are presented as percentage (%) or median (interquartile range).

^a^Chronic obstructive pulmonary disease, bronchiectasis, interstitial lung disease, parenchymal destruction associated with old inflammation, such as tuberculosis.

^b^The scoring system assigns 1 point for each of the following five risk factors: 1) new onset of confusion, 2) urea >7 mmol/l (19mg/dL), 3) respiratory rate ≥30 breaths/min, 4) systolic blood pressure <90 mm Hg and/or diastolic blood pressure ≤60 mm Hg and 5) age ≥65 years.

^c^A total point score for a given patient is obtained by adding the patient`s age in years (age minus 10, for women) and the points obtained for each applicable characteristic, such as nursing home resident, coexisting illness, physical examination findings, or laboratory and radiographic findings [22].

MDR = Multidrug resistance. SOFA = Sequential Organ Failure Assessment. PSI = Pneumonia Severity Index.

We investigated factors associated with mortality, according to origin of infection or culture positivity, as there was a difference in mortality rates. A similar trend was also found in significant risk factors compared with the total population. Common risk factors in all subgroups were: no use of intrapleural fibrinolytics, CURB-65 ≥ 2, SOFA score ≥ 2 and PSI risk class ≥ IV (Table 6).

**Table 6:** Univariate analysis for risk factors of 30-day mortality in patients with pleural infection according to origin of infection and culture positivity.

	30-day mortality							
Factors	Community acquired infection		Hospital acquired infection		Culture-negative		Culture-positive	
	Survival, n = 265	Death, n = 29	Survival, n = 96	Death, n = 31	Survival, n = 232	Death, n = 25	Survival, n = 129	Death, n = 35
Mortality rate (%)	-	(10.9)	-	(44.9)	-	(10.8)	-	(27.1)
Age, year	63 (51–74)	75 (71–78)**	66 (56–77)	65 (53–73)	64 (51–74)	71 (70–77)*	66 (57–74)	68 (54–77)
Male	214 (80.8)	23 (79.3)	66 (68.8)	25 (80.6)	180 (77.6)	18 (72.0)	100 (77.5)	30 (85.7)
Co-morbid conditions	193 (72.8)	25 (86.2)	84 (87.5)	26 (83.9)	171 (73.7)	21 (84.0)	106 (82.2)	30 (85.7)
Cardio-cerebrovascular disease	120 (45.3)	17 (58.6)	52 (54.2)	12 (38.7)	105 (45.3)	16 (64.0)	67 (51.9)	13 (37.1)
^a^Structural lung disease	40 (15.1)	11 (37.9))**	23 (24.0)	11 (35.5)	28 (12.1)	7 (28.0)*	35 (27.1)	15 (42.9)
Diabetes	65 (24.5)	10 (34.5)	31 (32.3)	10 (32.3)	58 (25.0)	7 (28.0)	38 (29.5)	13 (37.1)
Malignancy	23 (8.7)	4 (13.8)	8 (8.3)	4 (12.9)	16 (6.9)	3 (12.0)	15 (11.6)	5 (14.3)
Chronic kidney disease	22 (8.3)	5 (17.2)	16 (16.7)	4 (12.9)	20 (8.6)	5 (20.0)	18 (14.0)	4 (11.4)
Chronic liver disease	24 (9.1)	3 (10.3)	11 (11.5)	5 (16.1)	18 (7.8)	1 (4.0)	17 (13.2)	7 (20.0)
Ever-smoker	155 (58.5)	17 (58.6)	43 (44.8)	19 (61.3)	131 (56.5)	11 (44.0)	67 (51.9)	25 (71.4)*
Hospital-acquired infection	-	-	-	-	39 (16.8)	7 (28.0)	57 (44.2)	24 (68.6)*
Treatment								
Drainage only	197 (74.3)	26 (89.7)	62 (64.6)	22 (71.0)	168 (72.4)	22 (88.0)	91 (70.5)	26 (74.3)
Intrapleural fibrinolytics	97 (36.6))**	3 (10.3)	23 (24.0)*	2 (6.5)	77 (33.2)*	2 (8.0)	43 (33.3))**	3 (8.6)
Surgery	32 (12.1)	1 (3.4)	14 (14.6)	2 (6.5)	25 (10.8)	1 (4.0)	21 (16.3)	2 (5.7)
Antibiotics only	28 (10.6)	6 (20.7)	14 (14.6)	11 (35.5)*	29 (12.5)	9 (36.0))**	13 (10.1)	8 (22.9)*
Culture positivity	72 (27.2)	11 (37.9)	57 (59.4)	24 (77.4)	-	-	-	-
Pleural fluid characteristics								
Visibly purulent	159 (60.0)	17 (58.6)	35 (36.5)	10 (32.3)	133 (57.3)	11 (44.0)	61 (47.3)	16 (45.7)
pH	7.01 (6.70–7.14)	6.97 (6.56–7.13)	7.09 (6.84–7.30)	7.13 (6.96–7.26)	7.04 (6.82–7.15)	7.10 (6.85–7.13)	6.92 (6.64–7.23)	7.09 (6.85–7.23)
Leukocyte, /mm^3^	5184 (1097–24085)	3950 (606–17600)	3460 (886–20000)	5410 (1602–13665)	3100 (890–12440)	1916 (600–7040)	12765 (2512–75285)	8690 (1602–32900)
Glucose, mg/dL	36 (7–77)	55 (16–116)	36 (10–107)	60 (6–176)	43 (10–80)	56 (26–106)	27 (7–86)	46 (7–150)
Protein, g/dL	4.4 (3.1–5.0)	3.7 (3.2–4.7)	3.8 (2.6–4.4)	3.8 (3.0–4.8)	4.3 (3.1–4.9)	3.9 (3.1–4.8)	4.1 (2.6–5.0)	3.8 (3.1–4.8)
Lactate dehydrogenase, unit/L	2337 (1525–4561)	1789 (850–3353)	1907 (1059–6388)	2919 (1245–4870)	2232 (1505–4221)	1130 (639–3178)	2557 (1254–7550)	2971 (1637–5380)
^b^CURB-65 ≥ 2	94 (35.5)	27 (93.1))**	45 (46.9)	22 (71.0)*	82 (35.3)	21 (84.0))**	57 (44.2)	28 (80.0))**
SOFA score ≥ 2	99 (37.4)	20 (69.0))**	41 (42.7)	23 (74.2))**	102 (44.0)	20 (80.0))**	38 (29.5)	23 (65.7))**
^c^PSI risk class ≥ IV-V	124 (47.0)	26 (89.7))**	62 (64.6)	29 (93.5))**	115 (49.8)	22 (88.0))**	71 (55.0)	33 (94.3))**

Data are presented as percentages (%) or median (interquartile range).

^a^e.g, chronic obstructive pulmonary disease, bronchiectasis, interstitial lung disease, parenchymal destruction associated with old inflammation such as tuberculosis, etc.

^b^Scoring system assigns 1 point for each of the following five risk factors: 1) new onset confusion, 2) urea >7 mmol/l (19mg/dL), 3) respiratory rate ≥30 breaths/min, 4) systolic blood pressure <90 mm Hg and/or diastolic blood pressure ≤60 mm Hg and 5) age ≥65 years.

^c^A total point score for a given patient is obtained by adding the patient`s age in years (age minus 10, for women) and the points obtained for each applicable characteristic, such as nursing home resident, coexisting illness, physical examination findings, or laboratory and radiographic findings [22].

SOFA = Sequential Organ Failure Assessment. PSI = Pneumonia Severity Index.

** *p*<0.01

* *p*<0.05

In multivariate analysis using logistic regression, CURB-65 ≥2, structural lung disease, PSI risk class IV-V, no use of intrapleural fibrinolytics, hospital-acquired infection, older age and SOFA score ≥2 were identified as significant predictive factors for 30-day mortality in the total population (Table 7). SOFA score ≥2 was the most common factor in each subgroup, and antibiotic use was only added as a predictive factor in culture-negative patients with hospital-acquired infections.

**Table 7:** Multivariate analysis for risk factors of 30-day mortality in patients with pleural infection.

Factors	Total		Community acquired infection		Hospital acquired infection		Culture-negative		Culture-positive	
	Adjusted odds ratio (95% CI)	*p*	Adjusted odds ratio (95% CI)	*p*	Adjusted odds ratio (95% CI)	*p*	Adjusted odds ratio (95% CI)	*p*	Adjusted odds ratio (95% CI)	*p*
Age	0.964 (0.935–0.994)	0.018								
^a^CURB-65 ≥ 2	5.549 (2.296–13.407)	<0.001	18.611 (4.262–81.273)	<0.001	-	-	6.661 (2.146–20.672)	0.001		
SOFA score ≥ 2	2.361 (1.134–4.916)	0.022	3.005 (1.182–7.636)	0.021	3.047 (1.150–8.074)	0.025	4.286 (1.368–13.430)	0.013	3.901 (1.264–7.559)	0.013
^b^PSI risk class IV-V	4.714 (1.530–14.524)	0.007			5.276 (1.132–24.584)	0.034			7.903 (1.706–36.608)	0.008
Hospital acquired infection	2.205 (1.165–4.172)	0.015	NA	NA	NA	NA	-	-	-	-
No use of intrapleural fibrinolytics	3.062 (1.102–8.511)	0.014	-	-	-	-	-	-	4.714 (1.293–17.184)	0.019
^c^Structural lung disease	2.708 (1.364–5.379)	0.004	-	-	-	-	-	-	-	-
Antibiotic use only	-	-	-	-			2.817 (1.046–7.589)	0.041	-	-

^a^Scoring system assigns 1 point for each of the following five risk factors: 1) new onset confusion, 2) urea >7 mmol/l (19mg/dL), 3) respiratory rate ≥30 breaths/min, 4) systolic blood pressure <90 mm Hg and/or diastolic blood pressure ≤60 mm Hg and 5) age ≥65 years.

^b^A total point score for a given patient is obtained by summing the patient`s age in years (age minus 10, for women) and the points obtained for each applicable characteristic, such as nursing home resident, coexisting illness, physical examination findings, or laboratory and radiographic findings [22].

^c^e.g., chronic obstructive pulmonary disease, bronchiectasis, interstitial lung disease, parenchymal destruction associated with old inflammation such as tuberculosis, etc.

CI = Confidence Interval. NA = Not applicable. SOFA = Sequential Organ Failure Assessment. PSI = Pneumonia Severity Index.

## Discussion

The most frequent pathogens of pleural infection in this study were streptococci, in community-acquired infections, and *Staphylococcus aureus*, in hospital-acquired infections. Independent risk factors for 30-day mortality were CURB-65 ≥2, hospital-acquired infection, no use of intrapleural fibrinolytics, structural lung disease, and antibiotic use without intervention.

The first Multicenter Intrapleural Sepsis Trial (MIST1), found that common pathogens of community-acquired pleural infection were microorganisms of the *Streptococcus milleri* group (23.8%), *Streptococcus pneumoniae* (21.1%), and anaerobes (19.7%), and while in hospital-acquire infection, common pathogens were *Staphylococcus aureus* (35.0%), with MRSA (71.4%) infections occurring more frequently than methicillin-susceptible *Staphylococcus aureus* (28.6%), and gram-negative bacteria (23.3%) [4]. In a Danish multicenter study, the distribution of pathogens in pleural infection was similar to that observed in MIST1. Viridans streptococci (26%), anaerobic bacteria (20%), and *Staphylococcus aureus* (14%) were the most frequently isolated organisms in community-acquired infections, while *Staphylococcus aureus* (27%), viridans streptococci (20%), *Enterobacteriaceae* (20%), and anaerobic bacteria (14%) were the main organisms identified in hospital-acquired infections [8]. However, in a single center study of pleural infection pathogens in the United Kingdom (UK), a different distribution of isolated microorganisms was found: the most frequently isolated organisms were *Staphylococcus aureus* (15.5%), and *Streptococcus pneumonia* (9.6%), compared to microorganisms from the *Streptococcus milleri* group (4.2%) [9]. With the exception of our study, studies conducted in Asian countries found that pleural infection was associated with different pathogens compared to those identified in previous non-Asian studies (Table 8). In a study of non-surgical pleural infection at a single center in India, the most common pathogens were gram-negative bacteria, followed by gram-positive bacteria, among which *Staphylococcus aureus* was the most frequently isolated organism [12]. However, the study did not differentiate between organisms in community- and hospital-acquired infections. Another study in Taiwan found that gram-negative bacteria (57.7%) were the most common pathogens; however, this study was limited to patients admitted to the medical intensive care unit [3]. Two community-acquired infections were studied at single centers in Taiwan and Hong Kong [11, 15]. In these studies, Gram-negative pathogens were relatively common (34.5% and 31.6%) compared to our study, MIST1, the Danish study and the UK study, cited above. In Taiwan, two researches about hospital-acquired infections found that the most common pathogens were gram-negative organisms [13, 16]. These findings were not consistent with our study or previous studies where *Staphylococcus aureus* was the most common isolated pathogen in hospital-acquired infections [4, 8, 9]. With the exception of our study, Asian studies show that gram-negative pathogens are more common, both in community- and hospital-acquired pleural infection, than in our study, MIST1, the Danish study and the UK study [3, 11–13, 15, 16]. The cause for the different distribution of pathogens found in our study and previous Asian studies is not clear. The Asian studies, including our study, were conducted in a single center, and 4 out of 6 were conducted in a single center in Taiwan [3, 11–13, 15, 16]. Therefore, regional variations in pathogens associated with pleural infections may be the cause of these findings. The different culture systems used for evaluation of pathogens in pleural infection between our study and previous Asian studies may also account for these differences. Here, we used both blood culture and standard culture systems, whereas previous Asian studies only used the standard culture system.

**Table 8:** Comparison of microbiology in Asian studies.

Studies	This study		Malhotra et al.	Tu et al.	Liang et al.	Tsang et al.	Lin et al.	Chen et al.
Reference			10	3	14	11	12	15
Country	Korea		India	Taiwan	Taiwan	Hong Kong	Taiwan	Taiwan
Origin of infection	Community	Hospital	Community and hospital	Community and hospital	Community	Community	Hospital	Hospital
Hospital	Tertiary		Tertiary	Tertiary	Tertiary	Regional	Tertiary	Tertiary
Subjects (microorganisms)	83 (90)	81 (94)	55(72)	58(78)	46(55)	43 (57)	164 (225)	49(71)
Aerobes								
*Streptococcus*	47 (52.2%)	11 (11.7%)	7 (9.7%)	7 (9.0%)	21 (38.2%)	15 (26.3%)	35 (15.6%)	4 (5.6%)
*Streptococcus milleri* group (*intermedius/anginosus/constellatus*)	26	6	5		15	11		
Viridans streptococcus (other than *S*. *milleri*)	10	3				2		
*Streptococcus pneumoniae*	5	0	1		4	2		
*Staphylococcus*	7 (7.8%)	36 (38.3%)	10 (13.9%)	13 (16.7%)	5 (9.1%)	4 (7.0%)	28 (12.4%)	16 (22.5%)
MRSA	1	26	10	13		3	20	9
MSSA	4	5				1	8	7
*Enterococcus* species	1 (1.1%)	9 (9.6%)	1 (1.4%)	3 (3.8%)	1 (1.8%)	1 (1.8%)	9 (4.0%)	7 (9.9%)
Gram negatives	12 (13.3%)	22 (23.4%)	50 (69.4%)	45 (57.7%)	19 (34.5%)	18 (31.6%)	110 (48.9%)	38 (53.5%)
Anaerobes	14 (15.6%)	5 (5.3%)	4 (5.6%)	5 (6.4%)	9 (16.4%)	17 (29.8%)	34 (15.1%)	6 (8.5%)

Data are presented as numbers.

MRSA = methicillin-resistant *Staphylococcus aureus*; MSSA = methicillin-susceptible *Staphylococcus aureus*.

Unfortunately, few studies have evaluated pathogens of pleural infection in Korea [14, 17]. In a study of adult patients with pleural infection, pathogens were isolated in 31 out of 115 cases; alpha-hemolytic streptococci were the most common pathogen in nine patients (26%), followed by *Klebsiella pneumoniae* in 8 patients (26%) and *Staphylococcus aureus* in 5 patients (16%) [14]. However, this study enrolled a small number of patients with identifiable pathogens, and culture were obtained from sputum and blood, in addition to pleural fluid [14]. Furthermore, low levels of *Streptococcus pneumoniae* were identified in the present study (Tables 2 & 8). This may be the effect of the national public health project for pneumococcal vaccination, targeting the infant and elderly population in Korea. The majority of the elderly may have received the pneumococcal vaccination before enrolling in this study, although accurate histories of pneumococcal vaccination in individual patients were not known, due to the retrospective nature of this study. The number of MRSA isolates is also very high in this study, compared with previous Asian studies (Table 8). This finding could be explained by the fact that the study site is a tertiary referral center, with a high incidence of MRSA colonization (46%). Furthermore, nosocomial MRSA infections are detected in 10% of patients, at a rate of 8.0 per 1000 patient-days in this ICU, and MRSA was reported as the most common pathogen in ventilator associated pneumonia in this ICU[23, 24].

There are scarce data available on the differences between culture-positive and -negative pleural infection. One study investigated the efficacy of thoracotomy and decortication in patients with empyema, according to culture positivity [25]. Although all enrolled patients had community-acquired infection and were treated surgically, a subgroup, with culture positive empyema, had worse outcomes, in terms of a longer duration of pleural drainage, a longer duration of hospital stay, and more complications. In the present study, culture-positive patients had less favorable prognostic factors than those who were culture-negative. Culture-positive patients with pleural infection may have increased bacterial burden and higher severity. A trend of lower pH and glucose levels, and higher leukocytosis and lactate dehydrogenase levels, was observed. These findings could be associated with host fragility from pulmonary infection, possible due to comorbidities, such as chronic lung disease or liver disease. This may lead to a mortality gap between culture-positive and –negative subgroups, and even in total population.

As mentioned in the introduction and results sections of the present study, the microbiological findings in pleural infection are different to those in pneumonia, and the identification of the causative pathogen is difficult due to the low isolation rates in standard culture only. Therefore, antibiotics are generally given empirically, with no guidance from bacterial culture results, and, at this point, it is reasonable to follow guidelines for pleural infection, not community-acquired or hospital-acquired pneumonia. According to the 2010 BTS guidelines for pleural infection, antibiotics should cover both common community-acquired bacterial pathogens and anaerobic organisms, and MRSA and anaerobic bacteria should be considered when treating hospital-acquired empyema[6]. Therefore, in the present study, frequent use of a cephalosporin or a β-lactam/β-lactamase inhibitor combined with clindamycin, in community-acquired infection, and glycopeptides and colistin, in hospital-acquired infection, was considered adequate for the treatment of pleural infection. Antibiotic treatment showed favorable concordance, guided by susceptibility tests, particularly in community acquired infection (95.1%), when drugs were changed to the adequate regimen (78.1% to 90.7% in total). Discordant antibiotic treatment and delay in the use of the adequate antibiotic and drainage procedure, showed a difference according to origin of pleural infection, but they did not in mortality.

Mortality in pleural infection has been reported as ranging from 6 to 60% [3, 7–9, 11–13, 15, 16]. In our study, overall 30-day mortality rate was 14.3% (60/421). Among the risk factors for 30-day mortality, CURB-65≥2 was an independent predictor for death from pleural infection. CURB-65 has been used as a tool for the assessment of disease severity and the prediction of mortality in community-acquired pneumonia [26, 27]. Higher SOFA in patients with hospital-acquired infection indicated a more severe form of pleural infection and organ dysfunction, reflecting increase in risk of mortality. In a recent report on the assessment of clinical criteria for sepsis, patients in a general hospital population with a higher SOFA score, specifically of 2 or above, had an increased risk of overall mortality[28]. Another severity index of infection, a PSI score, identifies patients with pneumonia who are at low risk of death and other adverse outcomes, among three lowest risk classes in pneumonia patient outcomes research team cohort [22]. Interestingly, PSI risk class was a predictive factor for mortality in all patients, including subgroups of patients with hospital acquired infection, and culture-positive. The PIS score was a risk factor, not in community acquired infection but in hospital acquired infection, in contrast to CURB-65 ≥2 could not be explained. The initial assessment of a patient’s clinical situation by the severity score systems may be important in the effective treatment of pleural infection. The prompt drainage of pleural fluid is critical to the medical treatment of pleural infections. In our study, drainage procedures were performed in a majority of patients (350/421, 83.1%). This was associated with a favorable outcome of survival (310/361, 85.9% vs. 40/60, 66.7%, p = 0.001, data not shown in table). Antibiotic use without intervention, including a drainage procedure, could be a risk factor for death (Table 5). However, while drainage alone did not reach statistical significance, administration of intrapleural fibrinolytics was significantly associated with 30-day mortality in our study. The role of intrapleural fibrinolytic treatment in pleural infection remains debatable, mainly due to the results of large randomized studies, including MIST1 and MIST2 [29, 30]. However, a recent systematic review analyzed 7 randomized controlled studies, including MIST1 and MIST2, and showed that fibrinolytic treatment could be beneficial for the prevention of necessary surgical intervention or death [31]. Therefore, our findings were consistent with this meta-analysis.

This study found that hospital-acquired pleural infection was more likely to lead to death than community-acquired infection, as previous studies have shown [4, 9]. Patients with a hospital-acquired infection tended to have more comorbidities (86.6% vs. 74.1%, *p* = 0.005), such as structural lung disease (26.8% vs. 17.3%, *p* = 0.027), severe state of infection (CURB-65 ≥2; 52.8% vs. 41.2%, *p* = 0.028, SOFA score ≥2; 50.4% vs. 40.5%, *p* = 0.060, PSI risk class IV-V; 71.7% vs. 51.0%, *p*<0.001) and positive cultures (63.8% vs. 28.2%, p<0.001). Conversely, patients with community-acquired infections showed higher rates of visibly purulent fluid (59.9% vs. 35.4%, *p*<0.001), lower median pH (7.01 vs. 7.12, *p*<0.001), and lower median glucose levels (38.5 vs. 40.0 mg/dL, *p* = 0.029) than those with hospital-acquired infections. They also underwent more drainage procedures (75.9% vs. 66.1%, *p* = 0.040) or intrapleural fibrinolytic therapy (34.0% vs. 19.7%, *p* = 0.003) (data not shown in table). Therefore, patients with community-acquired infection may have more severe forms of the disease, but may have a more favorable outcome, in terms of mortality, than hospital-acquired infection, because of prompt medical intervention.

This study had several limitations. First, it was performed in a single center in South Korea. This may limit the generalizability of our findings for pathogens in pleural infection in South Korea or across broader areas. Second, not all pleural fluid specimens were cultured using both the standard method and BACTEC blood culture system. Therefore, different bacteria might be cultured preferentially in different culture systems.

In summary, the common pathogens of pleural infection in this study were streptococci, staphylococci and gram negatives. Predictive factors for mortality from this disease were CURB-65 ≥2, structural lung disease, PSI risk class IV-V, no use of intrapleural fibrinolytics, hospital-acquired infection, older age andSOFA score ≥ 2. Furthermore, antibiotic use without intervention could be an important risk factor for mortality in some subgroups.
